# A machine learning approach to predict ethnicity using personal name and census location in Canada

**DOI:** 10.1371/journal.pone.0241239

**Published:** 2020-11-18

**Authors:** Kai On Wong, Osmar R. Zaïane, Faith G. Davis, Yutaka Yasui

**Affiliations:** 1 School of Public Health, University of Alberta, Edmonton, Alberta, Canada; 2 Department of Computing Science, University of Alberta, Edmonton, Alberta, Canada; 3 Department of Epidemiology and Cancer Control, St. Jude Children’s Research Hospital, Memphis, Tennessee, United States of America; University of Oxford, UNITED KINGDOM

## Abstract

**Background:**

Canada is an ethnically-diverse country, yet its lack of ethnicity information in many large databases impedes effective population research and interventions. Automated ethnicity classification using machine learning has shown potential to address this data gap but its performance in Canada is largely unknown. This study conducted a large-scale machine learning framework to predict ethnicity using a novel set of name and census location features.

**Methods:**

Using census 1901, the multiclass and binary class classification machine learning pipelines were developed. The 13 ethnic categories examined were Aboriginal (First Nations, Métis, Inuit, and all-combined)), Chinese, English, French, Irish, Italian, Japanese, Russian, Scottish, and others. Machine learning algorithms included regularized logistic regression, C-support vector, and naïve Bayes classifiers. Name features consisted of the entire name string, substrings, double-metaphones, and various name-entity patterns, while location features consisted of the entire location string and substrings of province, district, and subdistrict. Predictive performance metrics included sensitivity, specificity, positive predictive value, negative predictive value, F1, Area Under the Curve for Receiver Operating Characteristic curve, and accuracy.

**Results:**

The census had 4,812,958 unique individuals. For multiclass classification, the highest performance achieved was 76% F1 and 91% accuracy. For binary classifications for Chinese, French, Italian, Japanese, Russian, and others, the F1 ranged 68–95% (median 87%). The lower performance for English, Irish, and Scottish (F1 ranged 63–67%) was likely due to their shared cultural and linguistic heritage. Adding census location features to the name-based models strongly improved the prediction in Aboriginal classification (F1 increased from 50% to 84%).

**Conclusions:**

The automated machine learning approach using only name and census location features can predict the ethnicity of Canadians with varying performance by specific ethnic categories.

## Introduction

Ethnicity and race are cornerstones of individuals’ sense of self-identity, social belonging, and shared experiences that influence one’s health beliefs, behaviours, and outcomes [1]. Ethnicity and race are socially-defined constructs that are complex and multilayered. While they are sometimes used interchangeably, they are two different but related concepts. The term “race” suggests a biological basis for socially-constructed categories that in-group members are implied to share greater genetic homogeneity than out-group members [2]. However, in reality, the degree of additional genetic similarity shared among members of the same race is largely negligible and biologically inconsequential compared to the total genetic makeup shared between individuals from different races [3]. The term “ethnicity” generally refers to a wide range of socially-constructed categories that in-group members tend to share a common culture, language, heritage, or national origin. While race is often characterized by a person’s physical attributes such as body height, hair texture, facial feature, and skin color, ethnicity is a person’s subjective affinity towards an ethnic group that he or she feels most self-identifiable with [4]. Since ethnicity is more widely used than race in Canada and it is conceptualized more narrowly for research and surveillance purposes [2], it is the focus of this research.

With great ethnic diversity, Canada is challenged by pervasive and persistent public health and social issues that are disproportionately affecting specific ethnic groups, including health inequality and racial discrimination towards Aboriginals and visible minorities [5, 6]. Specifically, Aboriginals experience significantly higher disease prevalence and worse clinical outcomes for a large number of acute and chronic conditions [7–9], yet they are also burdened by lower access and awareness to available health resources compared to non-Aboriginals in Canada [10–12]. As a result, ethnicity (and race) is considered a key social determinant of health in Canada [13]. Despite its public health significance and recognition, ethnicity data is absent in the vast majority of disease registries, patient-care administrative data, vital statistics data, and major health surveys across Canada [5, 6, 14]. This ethnicity data gap impedes the pursuit and attainment of ethnically-specific health evidence that are essential for the understanding, development, and monitoring of effective health policies and programs [5, 6].

Implementing large-scale, systematic data collection on ethnicity in health data across Canada may encounter many practical, political, and legal challenges [6, 15]. While the general public and health researchers and practitioners largely agree that collecting ethnicity for research is ethical and desirable, the net benefit may not be realized by all individuals [6]. For example, directly asking questions about ethnicity and race has evoked anxieties about racism and racist classification in some patients, especially those who have personally experienced racism in the healthcare system. Some interviewers have raised concerns regarding if the process of asking about ethnic and racial identities may steer the respondents to believe that inequality is endemic in the healthcare system [6]. An alternative approach that is timelier, less costly, and indirect in acquiring ethnicity information needs to be explored in Canada.

Unlike ethnicity, personal names are often collected in most databases by default. An alternative is to automate the prediction of ethnicity using commonly-collected variables such as personal names. Personal names are typically recorded in the form of unstructured format of the entire name or more structured format with specific name entities (such as first, middle, and last name). Since many naming practices are influenced by cultural, religious, and familial traditions that intersect with ethnicity, individuals’ first, middle, last, and full names carry a degree of predictive quality for the associated ethnicity. Mateos [16] conducted a systematic review which identified 13 representative studies that developed name-based ethnicity classification methodologies. The sensitivity and positive predictive value (PPV) of these studies ranged between 67–95% and 70–96%, respectively. These studies included a number of common methodological process and research components: 1) a name reference list is independently developed or sourced from another study or domain experts; 2) a separate target population (or testing data) is manually or automatically classified into different ethnic categories; and 3) the performance metrics of the method is evaluated against the previously known ethnicity (“gold standards”) in the target population [16]. The process of predicting or classifying ethnicity for the target population were either done manually or automatically. The manual approach typically involved domain experts examining and making the judgment of what the most likely ethnicity was for a particular name, based on the expertise in linguistic and ethnocultural history. In contrast, the automated approach did not rely on human judgment. Instead, it was based on programming the computer to automatically detect and utilize signals from names in the form of rule-based patterns (such as regular expression), statistical patterns (such as ML), or a combination of both. Mateos [16] found nine studies that have employed automated, as opposed to manual, ethnicity prediction methods to predict the ethnicity of the target population. Machine learning (ML) frameworks have continuously been explored in this area [17–20]. Ambekar et al. [17] combined decision tree and Hidden Markov Model (HMM) to conduct classification on a taxonomy with 13 ethnic categories. Treeratpituk et al. [15] examined both alphabetical and phonetic sequences in names to improve predictions. Fiscella and Fremont [21] and Imai and Khanna [22] have found that combining name and residence location further improved the performance.

Most published studies to date are non-Canadian with ML models trained with databases outside of Canada, which were not optimized for the Canadian population. Aboriginal populations must be included in this line of work as they are vastly marginalized and disadvantaged. In many ML publications, a formal procedure of feature selection and hyperparameter optimization may not be conducted or reported, thus reducing research reproducibility. Past Canadian studies using automated ML approaches to predict ethnicity tend to be scarce, outdated, examining only a few ethnic groups, and not including Aboriginals and their subgroups as distinct categories [16]. To our knowledge, no studies have combined textual and phonetic name features and location features to predict ethnicity. The primary objective of this study is to determine if the ethnicity data gap in Canada may be potentially addressed using a novel automated ML approach. This is achieved by conducting and formally evaluating the predictive performance of a large-scale ML framework utilizing a novel name and location feature set for ethnicity prediction. The secondary objectives of this study are to provide detailed description and to share codebase of this ML framework to support future research.

## Materials and methods

### Data source and analytic framework

**Fig 1:**
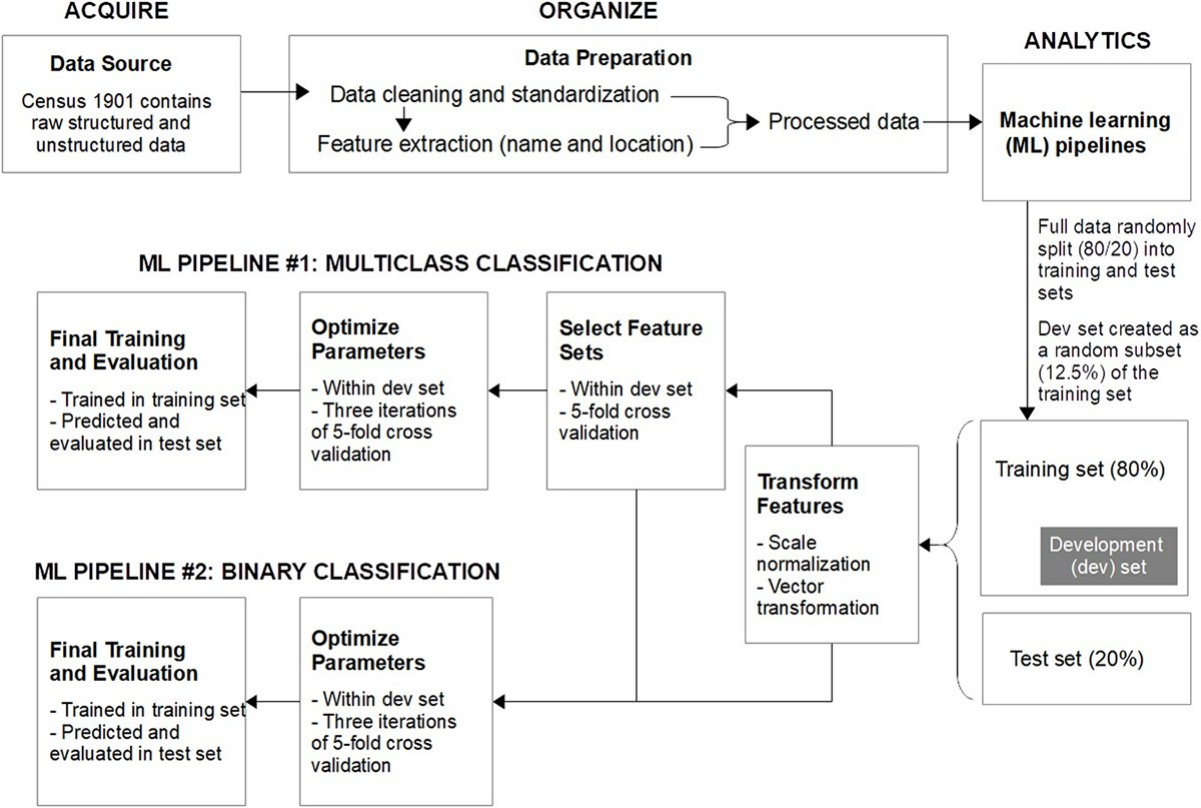
Overview of the machine learning framework.

The Canadian census contains all the required person-level variable fields, including self-reported full name, census location, sex, and ethnicity. From the Statistics Act of 2005, the release of personal records in past censuses is restricted for 92 years after their respective years of collection [23]. However, individual records from censuses prior to 1906 were publicly accessible at the National Archives of Canada. Under these constraints, we chose census 1901 as data source to build our ML framework (Fig 1). Features were extracted from the text recorded in name and census location variable fields. The census was split randomly into 80% training set and 20% test set. A development (dev) set was created by randomly-sampling 12.5% of the training set (Fig 1). Using only the dev set, the feature selection and hyperparameter optimization steps were conducted to finalize name and census location feature sets and to automatically optimize the ML algorithms, respectively. Finally, the obtained final feature sets and optimized hyperparameter values were used to train the ML algorithms within the training set. The trained ML algorithms would then be applied to predict the ethnicity of individuals in the test set. The agreement and discrepancy between the recorded ethnicity in census (as “gold standards”) and our predicted ethnicity were quantified based on a predefined set of evaluation metrics.

### Variables and initial data processing

ML models containing only sex or only dummy variables (with randomly-generated numeric or text strings) served as benchmarks, as they possessed no predictive quality for ethnicity. Incomplete (on name, census location, or ethnicity information) or duplicate records were removed. Name and ethnicity variables were in unstructured text format, thus they required further data cleaning and standardization. Personal titles, numbers, single alphabets, and punctuation marks were stripped from names. Alternative spellings and misspellings of ethnicity labels were standardized and recategorized into one of the followings: Aboriginal (Ab), English (En), Chinese (Ch), French (Fr), Irish (Ir), Italian (It), Japanese (Jp), Russian (Ru), Scottish (Sc), and others. These ethnic categories were selected either due to their large representation of the Canadian population (such as En, Sc, Fr, Ir, and It) or their public health and socioeconomic significance as ethnic minorities (such as Ab, Ch, and Jp), based on literature search and research team discussions. Some Aboriginals received a secondary label if they could be further categorized as First Nations (Ab-Fn), Métis (Ab-Mé), or Inuit (Ab-In).

The (census) location variable contained structured text of predefined census location information, including province/territory, district, and subdistrict, where the respondents were assigned to by Statistics Canada based on their residential addresses [24]. During the time, the major province/territory-level geographic boundaries included British Columbia, Manitoba, New Brunswick, Nova Scotia, Ontario, Prince Edward Island, Quebec, as well as Yukon Territory and Northwest Territories, and the District of Keewatin. One notable difference between this geographic categorization and current time is that Alberta and Saskatchewan were not considered a separate province but an individual district within the “Yukon Territory and Northwest Territories, and the District of Keewatin” region in census 1901 [24]. Nunavut was still part of the Northwest Territories until April 1^st^, 1999 [25], and Newfoundland and Labrador were not a Canadian province until its confederation on March 31, 1949 [26]. Census geographic boundaries did change over time due to major political events and population growth. However, boundary revisions rarely occurred and were only done when necessary, in order to maintain high comparability between censuses [27]. Thus, we believe the census location from census 1901 has retained useful and representative information, and the predictive quality of the underlying ML methodology remains highly relevant to current time.

### Feature engineering

The feature extraction process to create individual and grouped name features are described in Table 1, using “Wing Sun Lee” as an example. A technical challenge using the name variable in census 1901 was that it contained unstructured text, as opposed to the more recent censuses that divided the name into “given name” and “family name” at data collection. We labeled the first name entity (“Wing”) as “First name”, the last name entity (“Lee”) as “Last name”, and any remaining text string as “Middle name”. While we recognize that the most accurate split of the Chinese name “Wing Sun Lee” should divide “Wing Sun” as first name and “Lee” as last name, this name splitting would require the foreknowledge that it belongs to a Chinese person. Despite these exceptions, our selected name splitting method should be generalizable to most individuals in most databases where the information of ethnicity is unavailable. The key aspect was that all the textural information of the name was captured from the span of the extracted name features as a whole, even though there would be a small information lost due to the incorrect placement into the “First name”, “Middle name”, and “Last name” predictor slots for a small number of individuals.

**Table 1:** Extraction and groupings of name features.

		Full name	First name	Middle name	Last name
		(“Wing Sun Lee”)	(“Wing”)	(“Sun”)	(“Lee”)
**Basic name features**	**Individual name entities**	“wing”, “sun”, “lee”	n/a	n/a	n/a
**Name substring features**	**First letter**	n/a	“w”	“s”	“l”
	**Last letter**	n/a	“g”	“n”	“e”
	**1-letter substrings**	“w”, “i”, “n”, “g”, “s”, “u”, “l”, “e”	“w”, “i”, “n”, “g”	“s”, “u”, “n”	“l”, “e”
	**2-letter substrings**	n/a	“wi”, “in”, “ng”	“su”, “un”	“le”, “ee”
	**3-letter substrings**	n/a	“win”, “ing”	“sun”	“lee”
	**4-letter substrings**	n/a	“wing”	None	None
	**5-letter substrings**	n/a	None	None	None
	**6-letter substrings**	n/a	None	None	None
**Phonetic name features**	**Phonemes using double-metaphone algorithms [28]**	“ANK”, “FNK”, “SN”, “L”	n/a	n/a	n/a
**Numeric name features**	**Number of name entity**	3 (“wing”, “sun”, “lee”)	n/a	n/a	n/a
	**Total character length**	4 (“wing”) +3 (“sun”) +3 (“lee”) = 10	n/a	n/a	n/a
	**Average character length by name entity**	(4+3+3)/3 = 3.3	n/a	n/a	n/a
	**Number of vowels**	4 (“i”, “u”, “e”, “e”)	n/a	n/a	n/a
	**Vowel-to-length ratio**	4/10 = 0.4	n/a	n/a	n/a

n/a, not applicable.

For the location features, in addition to the existing full location string values, the corresponding province/territory, district, and subdistrict were extracted into three separate features. For example, the original location string of “H, L'Assomption, Quebec, Canada” corresponds to “subdistrict number, district, province/territory, country”. The text was processed by removing the non-informative “Canada” and lowercasing into “h, l'assomption, quebec” (as “Full location string”). It was then further broken into individual features as “quebec” (as “Province/Territory”), “l’assomption” (as “District”), and “h” (as “Subdistrict”). “All location features” included all four location features “Full location string”, “Province/Territory”, “District”, and “Subdistrict”.

### Machine learning pipelines

Our ML framework consisted of two pipelines (Fig 1). The fundamental difference between them was that in the multiclass classification pipeline, the predicted ethnicity was one of the 10 ethnicity labels, whereas in the binary classification pipeline, in each iteration, the original ethnicities were recategorized into a binary label (i.e., Ab or non-Ab). The multiclass classification pipeline involved the following steps: feature transformation, feature set selection, hyperparameter optimization, and final training and testing (Fig 1). Both feature set selection and hyperparameter optimization were based on k-fold cross validation (CV) conducted within the dev set. The k-fold CV proceeded the following sequentially: shuffling the data randomly, splitting data into k groups, and iterating the training/testing in each group for k times. Iteratively, the training and testing in each group involved taking one of the k groups as a test set, while the remaining groups as training set, and fitting ML model on the training set and evaluating it on the test set separately. Feature set selection was done by the a-priori criteria set by our research team. In order to tune the ML algorithms to optimize their learning prior to the final training and testing, the hyperparameter optimization step automatically selected the hyperparameter values corresponding to the highest F1-score (F1) for each ML algorithm. This approach was chosen since 1) the ML algorithms would be expected to learn from our data more effectively, and 2) the lack of previously-published Canadian studies precluded us to choose the final feature sets and hyperparameter values in a more manual fashion.

For feature transformation, all numeric features were scaled to zero mean and unit variance. Categorical features containing a single string value per individual were encoded as one-hot numeric arrays. Categorical features containing multiple string values (such as 1- to 6-letter substrings and double-metaphones) per individual were converted to matrices of token counts, known as count vectorization. For feature set selection, five-fold CV within dev set with regularized logistic regression (LR) classifiers was done. A-priori decision was made to derive two final feature sets: 1) “All name features” and 2) “All name and location features”. A-priori feature inclusion criteria and steps were used to confirm the inclusion of individual name features. “Basic name features”, “Name substring features”, “Numeric name features”, and “Phonetic name features” would only be included in the final “All name features” if its F1 was greater than the F1 of benchmarks by at least 10% (dummy feature as denominator). “All location features” were added to the “All name features” to create the “All name and location features”. As a confirmation, it was expected that the “All name and location features” would outperform the “All name features” by at least 10% in F1 (“All name features” as denominator). Regardless if this confirmation was actually observed in data, the “All name and location features” would proceed in both ML pipelines, since it was expected that the addition of location features would at least boost the predictive performance for some of the individual ethnic categories in binary classifications. We assumed that creating an individualized feature set for each binary ethnic category would not significantly improve the predictive performance. Thus, the final feature sets obtained from the multiclass classification pipeline would also be applied to the binary classification pipeline (Fig 1).

The ML hyperparameter optimization was done only in the dev set to determine hyperparameter values that maximized F1. ML classifiers including the regularized LR, C-support vector (SVC), naïve Bayes (NB), decision trees (DT), and random forest (RF) were implemented. Randomized search via 5-fold CV repeated three times to obtain the highest F1 was done for each ML algorithm (Fig 1). The final feature sets and optimized hyperparameters were then used in the final training and testing step. Training was done in the training set, and the trained models were applied to predict individuals’ ethnicity in the unseen (or hold-out) test dataset. The evaluation metrices on predictive performance consisted of accuracy, sensitivity, specificity, PPV, negative predictive value (NPV), F1, Area Under the Curve for Receiver Operating Characteristic curve (AUC-ROC), and average PPV [29]. AUC-ROC and average PPV are indicators that summarize how well the ML classifiers perform over a range of thresholds for decision boundary [30].

All data cleaning and preprocessing, descriptive analysis, ML pipelines, and visualization were carried out in Python 3.6.5 and related scientific libraries (such as Pandas, Numpy, Scikit-Learn, Statsmodels, and Metaphone). The codebase was made publicly available as a GitHub repository at https://github.com/kaionwong/ethnicity-ml-prediction.

## Results

**Fig 2:**
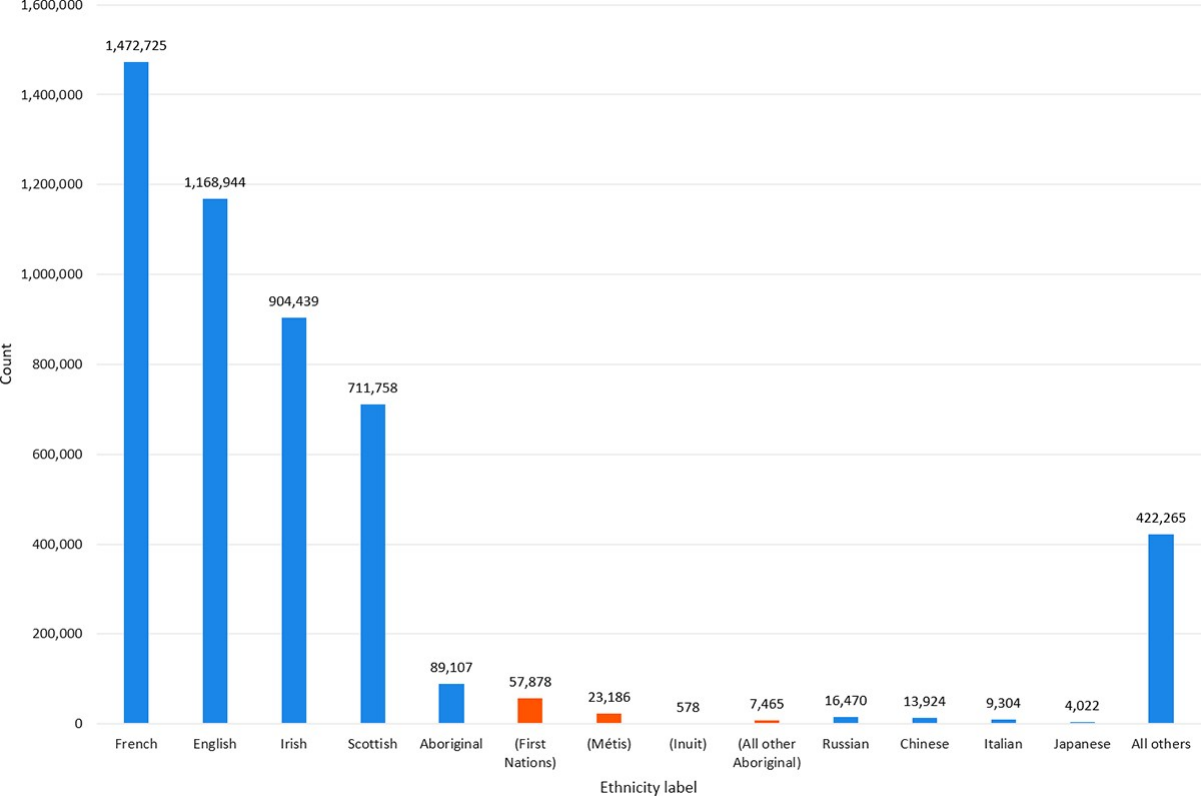
Frequency count breakdown by ethnic groups in census 1901 (N = 4,812,958). Each Aboriginal person represents two counts: one in the Aboriginal label and another in one of the followings: First Nations, Métis, Inuit, or all-other-Aboriginal label.

Census 1901 initially contained 5,079,210 records. After removing missing and duplicated records, the total number of unique individuals was 4,812,958 (94.8%). From this, 20% randomly extracted as test set (N = 962,592). The remaining 80% became training set (N = 3,850,366). 12.5% of the training set was randomly-selected to form the dev set (N = 481,296). The breakdown by ethnicity is illustrated in Fig 2. Considerable class imbalance was observed in some of the minority ethnic categories (i.e., Inuit) with low occurrence frequencies.

### Feature selection

Table 2 shows that all the name and location features outperformed the benchmarks by at least 10% in F1, fulfilling the a-priori criteria for inclusion. The “All name features” outperformed all the individual name feature subsets by 7% to 275% in F1. The “All name and location features” outperformed the “All name features” and “All location features” by 20% and 76%, respectively. The content of the “All name features” and “All name and location features” were confirmed, as previously described, to be the final feature sets for both ML pipelines.

**Table 2:** Feature set selection result for multiclass classification using 5-fold cross-validation in development set.

Feature set	Sensitivity	PPV	F1-score
**Dummy features only (as benchmark)**	0.10	0.03	0.05
**Sex only (as benchmark)**	0.11	0.08	0.06
**Basic name features (only)**	0.46	0.69	0.51
**Name substring features (only)**	0.50	0.71	0.56
**Numeric name features (only)**	0.19	0.20	0.16
**Phonetic name features (only)**	0.29	0.51	0.31
**All name features (chosen as a final set)**^**a**^	0.55	0.72	0.60
**All location features**	0.39	0.48	0.41
**All name and location features (chosen as a final set)**^**a**^	0.68	0.79	0.72

PPV, positive predictive value. Dummy features only = randomly-generated string feature and randomly-generated numeric feature. Basic name features only = first, middle, and last names and their corresponding first and last characters. Name substring features only = 1- to 6-letter strings for first, middle, and last names. Numeric name features only = number of name entities, total character length, total character length by name entity, number of vowels, and vowel-to-length ratio. All name features = all name-derived features. All location features = all location-derived features including processed location text string, province/territory, district, and sub-district features. All name and location features = “All name features” and “All location features”.

^a^The two chosen final feature sets “All name features” and “All name and location features” were then passed down to the subsequent steps for both multiclass and binary classification pipelines.

### Multiclass classification

Table 3 describes the predictive performance for multiclass classification. The DT and RF classifiers consistently and vastly underperformed compared to LR, SVC, and NB classifiers, thus their results were omitted. Overall, LR classifiers tended to perform marginally better than SVC, while both were more superior over NB. The “All name and location features” expectedly achieved the best overall performance. Improvement by adding the location features for specific ethnicity label varied widely between 2% (Ch and Fr) and 34% (Ab) in F1 for the LR classifiers.

**Table 3:** Multiclass classification predictive performance in test set with ML models trained in training set.

Feature set	ML algorithm	Ethnicity	Sensitivity	Specificity	PPV	NPV	F1-score	Accuracy
Dummy features; Sex	LR, SVC, NB	Overall	0.31	0.69	0.12	0.79	0.14	0.86
**All name features**	**LR**	**Ab**	0.38	1.00	0.76	0.99	0.50	0.99
		**Ch**	0.89	1.00	0.94	1.00	0.92	1.00
		**En**	0.74	0.83	0.59	0.91	0.65	0.81
		**Fr**	0.94	0.96	0.91	0.97	0.93	0.95
		**Ir**	0.54	0.93	0.63	0.90	0.58	0.85
		**It**	0.49	1.00	0.80	1.00	0.61	1.00
		**Jp**	0.72	1.00	0.87	1.00	0.79	1.00
		**Others**	0.54	0.98	0.70	0.96	0.61	0.94
		**Ru**	0.67	1.00	0.88	1.00	0.76	1.00
		**Sc**	0.60	0.95	0.66	0.93	0.63	0.90
		**Overall**^**a**^	0.72	0.92	0.72	0.94	0.71	0.89
	**SVC**	**Overall**^**a**^	0.71	0.93	0.71	0.93	0.71	0.89
	**NB**	**Overall**^**a**^	0.67	0.93	0.69	0.93	0.67	0.88
**All name and location features**	**LR**	**Ab**	0.82	1.00	0.86	1.00	0.84	0.99
		**Ch**	0.92	1.00	0.95	1.00	0.94	1.00
		**En**	0.76	0.86	0.64	0.92	0.69	0.94
		**Fr**	0.95	0.97	0.94	0.98	0.95	0.97
		**Ir**	0.61	0.93	0.67	0.91	0.64	0.87
		**It**	0.57	1.00	0.83	1.00	0.67	1.00
		**Jp**	0.86	1.00	0.94	1.00	0.90	1.00
		**Others**	0.59	0.98	0.74	0.96	0.66	0.95
		**Ru**	0.78	1.00	0.91	1.00	0.84	1.00
		**Sc**	0.63	0.95	0.70	0.94	0.66	0.90
		**Overall**^**a**^	0.76	0.94	0.76	0.94	0.76	0.91
	**SVC**	**Overall**^**a**^	0.75	0.94	0.76	0.94	0.75	0.91
	**NB**	**Overall**^**a**^	0.69	0.93	0.70	0.93	0.70	0.89

ML, machine learning; PPV, positive predictive value; NPV, negative predictive value; LR, regularised logistic regression classifier; SVC, C-support vector classifier; NB, naïve Bayes classifier.

^a^Weighted average across individual ethnic categories.

The multiclass confusion matrix for “All name and location features” with LR is shown in Table 4. The multiclass confusion matrix for “All name features” with LR is shown in Table 5.

**Table 4:** Multiclass confusion matrix in test set for the “All name and location features” set trained in training set with regularised logistic regression classifier.

		Predicted									
		Ab	Ch	En	Fr	Ir	It	Jp	Others	Ru	Sc
**Truth**^**a**^	**Ab**	14575	6	970	994	419	8	9	220	14	553
	**Ch**	16	2596	112	23	20	0	7	22	0	21
	**En**	539	34	176509	5697	26957	38	5	8002	45	15961
	**Fr**	821	3	6114	280663	3362	59	2	1877	13	1554
	**Ir**	315	20	43806	4455	110699	35	3	3693	19	18132
	**It**	17	1	193	401	80	1057	2	85	1	35
	**Jp**	8	18	34	5	6	4	678	25	2	10
	**Others**	313	31	20945	4188	5413	55	11	49759	145	3207
	**Ru**	15	1	147	47	53	2	2	443	2592	35
	**Sc**	352	10	28155	2405	18917	17	3	2721	5	89924

Ab, Aboriginal; Ch, Chinese; En, English; Fr, French; Ir, Irish; It, Italian; Jp, Japanese; Ru, Russian; Sc, Scottish.

^a^Color gradient is set across each row with darker green indicating higher counts.

**Table 5:** Multiclass confusion matrix in test set for the “All name features” set trained in training set with regularised logistic regression classifier.

		Predicted									
		Ab	Ch	En	Fr	Ir	It	Jp	Others	Ru	Sc
**Truth**^**a**^	**Ab**	6686	47	3975	2875	1448	13	37	942	18	1727
	**Ch**	64	2515	99	56	33	0	5	25	0	20
	**En**	516	27	172172	7881	27580	38	7	8729	34	16803
	**Fr**	468	10	9116	276346	3965	82	9	2489	22	1961
	**Ir**	333	14	52884	5263	98675	27	7	3654	15	20305
	**It**	20	1	186	524	82	922	4	87	1	45
	**Jp**	64	12	30	50	17	4	565	34	2	12
	**Others**	339	32	23599	6106	5171	48	11	45207	196	3358
	**Ru**	16	0	203	126	65	3	1	650	2234	39
	**Sc**	260	10	31015	2986	19495	12	3	2709	14	86005

Ab, Aboriginal; Ch, Chinese; En, English; Fr, French; Ir, Irish; It, Italian; Jp, Japanese; Ru, Russian; Sc, Scottish.

^a^Color gradient is set across each row with darker green indicating higher counts.

### Binary class classification

The binary classification predictive performance is described in Table 6 for LR. Similar tables for SVR and NB are presented in Tables 7 and 8, respectively. The addition of location features improved markedly for specific groups (i.e., more than 10% increase in F1 for Irish, Japanese, others, and all Aboriginal groups) (Table 6). For the “All name and location features”, 7 out of 13 (53.8%) and 10 out of 13 (76.9%) binary ethnicity classifications achieved 70% or greater in F1 and average PPV, respectively (Table 6). The accuracy and AUC-ROC ranged from 85–100% and 82–100%, respectively.

**Table 6:** Binary classification predictive performance in test set based on regularised logistic regression classifiers trained in training set.

Feature set	Ethnicity	Sensitivity	Specificity	PPV	NPV	F1-score	AUC-ROC	Average PPV	Accuracy
Dummy features; Sex	Across all binary label pairs	0	1.00	N/A	0.69–1.00	N/A	0.50–0.54	0–0.31	0.69–1.00
**All name features**	**Ab**	0.36	1.00	0.80	0.99	0.50	0.88	0.50	0.99
	**Ab-Fn**	0.35	1.00	0.82	0.99	0.49	0.92	0.52	0.99
	**Ab-Mé**	0.20	1.00	0.69	1.00	0.31	0.89	0.30	1.00
	**Ab-In**	0.08	1.00	0.13	1.00	0.10	0.88	0.07	1.00
	**Ch**	0.88	1.00	0.96	1.00	0.92	1.00	0.95	1.00
	**En**	0.84	0.78	0.55	0.94	0.67	0.89	0.71	0.80
	**Fr**	0.92	0.98	0.95	0.97	0.94	0.99	0.97	0.96
	**Ir**	0.48	0.96	0.72	0.89	0.57	0.88	0.67	0.87
	**It**	0.50	1.00	0.81	1.00	0.62	0.95	0.59	1.00
	**Jp**	0.67	1.00	0.88	1.00	0.76	0.99	0.80	1.00
	**Others**	0.51	0.99	0.81	0.96	0.63	0.91	0.69	0.95
	**Ru**	0.72	1.00	0.90	1.00	0.80	0.98	0.81	1.00
	**Sc**	0.53	0.97	0.74	0.92	0.62	0.90	0.68	0.90
**All name and location features**	**Ab**	0.80	1.00	0.88	1.00	0.84	0.99	0.90	0.99
	**Ab-Fn**	0.78	1.00	0.84	1.00	0.81	1.00	0.88	1.00
	**Ab-Mé**	0.56	1.00	0.77	1.00	0.65	0.99	0.67	1.00
	**Ab-In**	0.57	1.00	0.22	1.00	0.31	1.00	0.35	1.00
	**Ch**	0.89	1.00	0.96	1.00	0.93	1.00	0.96	1.00
	**En**	0.63	0.92	0.72	0.89	0.67	0.91	0.88	0.85
	**Fr**	0.96	0.98	0.95	0.98	0.95	0.99	0.99	0.97
	**Ir**	0.55	0.96	0.74	0.90	0.63	0.91	0.73	0.88
	**It**	0.58	1.00	0.83	1.00	0.68	0.98	0.69	1.00
	**Jp**	0.82	1.00	0.91	1.00	0.87	1.00	0.91	1.00
	**Others**	0.61	0.99	0.82	0.96	0.70	0.94	0.77	0.95
	**Ru**	0.81	1.00	0.92	1.00	0.86	0.99	0.89	1.00
	**Sc**	0.57	0.97	0.76	0.93	0.65	0.82	0.74	0.91

ML, machine learning; PPV, positive predictive value; NPV, negative predictive value; AUC-ROC, area under the curve for receiver operating characteristic curve; Ab, Aboriginal; Ab-Fn, First Nations; Ab-Mé, Métis; Ab-In, Inuit; Ch, Chinese; En, English; Fr, French; Ir, Irish; It, Italian; Jp, Japanese; Ru, Russian; Sc, Scottish; N/A, can not be calculated.

**Table 7:** Binary classification predictive performance in test set based on C-support vector classifiers trained in training set.

Feature set	Ethnicity	Sensitivity	Specificity	PPV	NPV	F1-score	AUC-ROC	Average PPV	Accuracy
Dummy features; Sex	Across all binary label pairs	0	1.00	N/A	0.69–1.00	N/A	0.50–0.54	0–0.31	0.69–1.00
**All name features**	**Ab**	0.35	1.00	0.86	0.99	0.50	0.90	0.52	0.99
	**Ab-Fn**	0.35	1.00	0.88	0.99	0.50	0.90	0.51	0.99
	**Ab-Mé**	0.00	1.00	0.90	1.00	0.00	0.87	0.19	1.00
	**Ab-In**	0.03	1.00	1.00	1.00	0.06	0.92	0.16	1.00
	**Ch**	0.87	1.00	0.96	1.00	0.92	1.00	0.94	1.00
	**En**	0.40	0.94	0.67	0.83	0.50	0.85	0.63	0.81
	**Fr**	0.91	0.98	0.95	0.96	0.93	0.98	0.97	0.96
	**Ir**	0.46	0.96	0.74	0.89	0.57	0.88	0.68	0.87
	**It**	0.42	1.00	0.88	1.00	0.57	0.95	0.61	1.00
	**Jp**	0.64	1.00	0.88	1.00	0.74	0.99	0.79	1.00
	**Others**	0.52	0.99	0.81	0.96	0.63	0.91	0.69	0.95
	**Ru**	0.67	1.00	0.91	1.00	0.77	0.98	0.78	1.00
	**Sc**	0.52	0.97	0.76	0.92	0.62	0.91	0.70	0.90
**All name and location features**	**Ab**	0.77	1.00	0.89	1.00	0.83	0.99	0.90	0.99
	**Ab-Fn**	0.75	1.00	0.82	1.00	0.78	0.99	0.84	1.00
	**Ab-Mé**	0.48	1.00	0.83	1.00	0.61	0.98	0.64	1.00
	**Ab-In**	0.00	1.00	N/A	1.00	N/A	0.00	0.00	1.00
	**Ch**	0.90	1.00	0.97	1.00	0.93	1.00	0.96	1.00
	**En**	0.66	0.91	0.71	0.89	0.68	0.91	0.74	0.85
	**Fr**	0.95	0.99	0.96	0.98	0.96	0.99	0.99	0.97
	**Ir**	0.52	0.96	0.76	0.90	0.62	0.91	0.72	0.88
	**It**	1.00	1.00	0.88	1.00	0.66	0.98	0.72	1.00
	**Jp**	0.70	1.00	0.94	1.00	0.81	1.00	0.89	1.00
	**Others**	0.57	0.99	0.83	0.96	0.68	0.94	0.76	0.95
	**Ru**	0.77	1.00	0.94	1.00	0.85	0.99	0.89	1.00
	**Sc**	0.56	0.97	0.78	0.93	0.65	0.92	0.74	0.91

ML, machine learning; PPV, positive predictive value; NPV, negative predictive value; AUC-ROC, area under the curve for receiver operating characteristic curve; Ab, Aboriginal; Ab-Fn, First Nations; Ab-Mé, Métis; Ab-In, Inuit; Ch, Chinese; En, English; Fr, French; Ir, Irish; It, Italian; Jp, Japanese; Ru, Russian; Sc, Scottish; N/A, can not be calculated.

**Table 8:** Binary classification predictive performance in test set based on naïve Bayes classifiers trained in training set.

Feature set	Ethnicity	Sensitivity	Specificity	PPV	NPV	F1-score	AUC-ROC	Average PPV	Accuracy
Dummy features; Sex	Across all binary label pairs	0	1.00	N/A	0.69–1.00	N/A	0.50–0.54	0–0.31	0.69–1.00
**All name features**	**Ab**	0.70	0.84	0.08	0.99	0.14	0.85	0.23	0.84
	**Ab-Fn**	0.70	0.91	0.09	1.00	0.16	0.89	0.32	0.91
	**Ab-Mé**	0.46	0.95	0.04	1.00	0.08	0.85	0.05	0.95
	**Ab-In**	0.60	0.99	0.01	1.00	0.01	0.92	0.01	0.99
	**Ch**	0.95	1.00	0.44	1.00	0.61	0.99	0.86	1.00
	**En**	0.87	0.67	0.46	0.94	0.60	0.84	0.58	0.72
	**Fr**	0.92	0.96	0.90	0.96	0.91	0.97	0.94	0.94
	**Ir**	0.81	0.71	0.39	0.94	0.52	0.84	0.55	0.72
	**It**	0.78	0.96	0.03	1.00	0.06	0.94	0.17	0.96
	**Jp**	0.92	0.99	0.10	1.00	0.19	0.99	0.55	0.99
	**Others**	0.78	0.81	0.29	0.98	0.42	0.88	0.54	0.81
	**Ru**	0.81	0.98	0.11	1.00	0.20	0.97	0.31	0.98
	**Sc**	0.76	0.82	0.43	0.95	0.55	0.88	0.59	0.81
**All name and location features**	**Ab**	0.77	0.96	0.29	1.00	0.42	0.95	0.60	0.96
	**Ab-Fn**	0.79	0.97	0.27	1.00	0.40	0.96	0.59	0.97
	**Ab-Mé**	0.68	0.97	0.09	1.00	0.16	0.93	0.17	0.97
	**Ab-In**	0.67	0.99	0.01	1.00	0.02	0.94	0.02	0.99
	**Ch**	0.95	1.00	0.54	1.00	0.69	0.99	0.89	1.00
	**En**	0.86	0.70	0.48	0.94	0.61	0.85	0.60	0.74
	**Fr**	0.92	0.96	0.92	0.97	0.92	0.98	0.95	0.95
	**Ir**	0.77	0.73	0.40	0.93	0.52	0.83	0.54	0.74
	**It**	0.72	0.99	0.09	1.00	0.17	0.94	0.31	0.99
	**Jp**	0.92	1.00	0.16	1.00	0.27	0.99	0.68	1.00
	**Others**	0.79	0.84	0.32	0.98	0.45	0.89	0.58	0.83
	**Ru**	0.87	0.99	0.20	1.00	0.32	0.98	0.50	0.99
	**Sc**	0.72	0.85	0.46	0.95	0.56	0.88	0.59	0.83

ML, machine learning; PPV, positive predictive value; NPV, negative predictive value; AUC-ROC, area under the curve for receiver operating characteristic curve; Ab, Aboriginal; Ab-Fn, First Nations; Ab-Mé, Métis; Ab-In, Inuit; Ch, Chinese; En, English; Fr, French; Ir, Irish; It, Italian; Jp, Japanese; Ru, Russian; Sc, Scottish; N/A, can not be calculated.

## Discussion

To our best knowledge, we conducted one of the most extensive ML research in Canada within the domain of ethnicity prediction using name and location information. Overall, we employed a two-pipeline approach to demonstrate our classifiers’ performance for a wider range of potential applications. The multiclass classifiers achieved 76% F1 and 91% accuracy. The confusion matrices showed that most frequently-misclassified labels for English, Irish, and Scottish individuals occurred among themselves. This is to be expected as these groups historically shared a large degree of cultural and linguistic heritage. To improve the performance, regrouping them all under one generic “British” category may be considered, as shown in Ambekar et al. [17].

Aboriginals are most frequently misclassified as En, Ir, Sc, and Fr. The Indian Act was first introduced in 1876 (prior to 1901 census) as a consolidation of previous colonial mandates and regulations that aimed to eradicate First Nations culture in favour of assimilation into Euro-Canadian societies [31]. Thus, the misclassifications among Aboriginals are likely a result of the Indian Act’s naming policies which unjustly forced Aboriginals to adopt new European names. Despite this inherent challenge, there was a large improvement in performance by adding location features onto the name feature sets. This aligned with the phenomenon that certain geographic regions in Canada were highly populated by Aboriginal populations [32]. For binary classifications, the sensitivity and F1 increased by 44% (36% to 80%) and 34% (50% to 84%) for “Aboriginal” predictions, and by 43% (35% to 78%) and 32% (49% to 81%) for “First Nations” predictions, respectively. Métis and Inuit binary classifications resulted in mediocre and poor predictions (65% and 31% F1, respectively), which is not unexpected due to the interracial marriage with Europeans among Métis [33] and the small available training data of Inuit in census data. Excluding the Ab, Ab-Fn, Ab-Mé, Ab-In, En, Ir, and Sc, the F1 ranged from 70% to 95% (median: 87%) in binary classification for the remaining six ethnic categories (Ch, Fr, It, Jp, Ru, Others). As mentioned in the Materials and Methods section, our name splitting method likely misplaced the first and last names from the correct name feature labels for a portion of Chinese individuals if their last names were recorded as the first (position) name entity. However, the consistently high predictive performance for Chinese (i.e., 0.88 sensitivity, 0.96 PPV, and 0.92 F1 in binary classification with “All name features” set) confirms our assumption that the information lost is negligible as long as all the name entities are considered and further extracted somewhere within the span of all the name features.

In Canada, the trends and degrees of urbanisation and migration differ by ethnic groups. From 1961 to 2006, the percent of Aboriginals living in urban areas (as opposed to Indian reserves and rural areas) increased from 13% to 53% [34]. More specifically from 1981 to 2006, it increased from 40% to 53% for Aboriginals and from 75% to 81% for non-Aboriginals. However, residential segregation by ethnicity or ethnicity-associated factors remains. For example, residential aggregation of visible minorities, recent immigrants, and Aboriginals exists and is found to be a key associative factor underpinning the increase of concentrated urban poverty in various regions [35]. In addition to being highly populated in Canadian territories (Northwest Territories, Yukon, and Nunavut), Aboriginals are found to become more concentrated in a number of Prairie cities, particularly Saskatoon, Regina, and Winnipeg in recent times. Families of visible minority account for up to 78% of the low-income families residing in high poverty neighborhoods in 2001, doubling the level in 1981 [35]. Despite the changes in geographic boundaries between censuses and the dynamic nature of residential mobility and migration by Canadians, these findings strongly suggest that discernable patterns in geographic segregation by specific ethnic groups may always exist. These serves as indirect evidence that our demonstrated ML method utilizing name and location features should remain relevant and applicable if it is to be adopted by modern datasets.

The feature selection step has shown alphabetic name features carry more predictive quality over phonetic name features, which aligned with findings by Treeratpituk and Giles [15]. Similar to Fiscella and Fremont [21] and Imai and Khanna [22], we found that combining name and location features markedly improved performance for some but not all ethnic categories. In terms of predictive performance, direct and complete comparison of our study to published studies is impossible since different studies have employed different methodologies (time periods, populations, data sources, ML classifiers, performance measures, and ethnicity labels and grouping). Nonetheless, a number of indirect comparisons are feasible. For the ethnic categories overlapped between our study and Ambekar et al. [17], we achieved better performance in French (our F1: 95%, their F1: 57%) and Japanese (our F1: 90%, their F1: 83%), and similar performance in Italian (our F1: 67%, their F1: 66%). For the ethnic categories overlapped between our and Treeratpituk and Giles [15], we achieved better performance in French (our F1: 95%, their F1: 80%), Russian (our F1: 84%, their F1: 81%), and Chinese (our F1: 94%, their F1: 91%), but worse performance in Italian (our F1: 67%, their F1: 85%) and Japanese (our F1: 90%, their F1: 96%). For previous Canadian studies, our Chinese classifier (92% sensitivity, 95% PPV, 94% F1) outperformed Choi et al. (1993) (73% sensitivity, 81–84% PPV) [36] and is comparable to Coldman et al. (1988) (89–97% sensitivity) [37].

In terms of potential real-life applications, the trained ML classifiers using “All name features” can be applied to the personal name field of other databases to generate predicted ethnicity at the individual-level. However, databases that also contain respondents’ residential addresses will not be able to use our trained ML classifiers with “All name and location features” directly since they are trained with the older (1901) census boundaries. An additional step is needed to standardize and remap the location information before using both name and location information from applied databases. For example, one option is to convert both the location information in both census 1901 and applied database to the Global Positioning System (GPS) coordinates. The individuals in the applied database will receive an approximated census location, via mapping the GPS of their addresses to the nearest corresponding census location, which can then be used directly by our trained ML classifiers. Available remapping tools exist that enable the geographic conversion of residential addresses to corresponding census location information [38].

### Limitations

This study used an older Canadian census 1901, which is about four generations (30 years per generation) from the past. Studies have shown that distinctive naming practices in different ethnic groups are often persistent over a long period of time, even after immigration to another geographic location with different cultural and social environment [39–41]. As mentioned earlier, despite continuous urbanisation and migration, distinctive residential segregation patterns exist by different ethnic groups. As a result, we believe the underlying ML methodology conducted is applicable and generalizable to more recent time. Nonetheless, we encourage future studies with similar research interest to access and evaluate with a more recent data source to strengthen the temporal representativeness of the ML models. We also recommend future studies to expand on the current ethnic categories, as well as examine regrouping ethnicity labels into hierarchical structures that is relevant in the Canadian context.

## Conclusions

This is the first comprehensive Canadian study to show that a wide range of ethnic categories can be accurately predicted using a ML framework that learns from relatively simple and widely-collected personal name and location information. There are many potential public health applications (i.e., disease and risk factor surveillance, effectiveness of intervention, and patterns in health service utilization and related costs) in which adding the ethnicity dimension will greatly multiply the value, utility, and relevance of the existing information. Wide-spread implementation of ethnicity classifiers will help generate ethnically-specific health evidence that together will fill many critical knowledge gaps that currently impede effective health program and policy development in Canada.
